# Optimization of hormone combinations for root growth and bud germination in Chinese fir (*Cunninghamia lanceolata*) clone leaf cuttings

**DOI:** 10.1038/s41598-017-05295-z

**Published:** 2017-07-11

**Authors:** Shubin Li, Peng Huang, Guochang Ding, Lili Zhou, Piao Tang, Min Sun, Yingying Zheng, Sizu Lin

**Affiliations:** 1 Forestry College, Fujian Agriculture and Forestry University, Fuzhou, 350002 P. R. China; 2 Chinese Fir Engineering Technology Research Center of the State Forestry Administration, Fuzhou, 350002 P. R. China; 3 Zhejiang Lishui High School, Lishui, 323000 P. R. China; 4 College of Arts College of Landscape Architecture, Fujian Agriculture and Forestry University, Fuzhou, 350002 P. R. China; 5 The Institute of Oceanography, Minjiang University, Fuzhou, 350108 P. R. China

## Abstract

In order to ascertain the optimal hormone combination for Chinese fir (*Cunninghamia lanceolata*) leaf cuttings, an L_16_(4)^4^ orthogonal test of 6-benzylaminopurine (6-BA), 1-naphthaleneacetic acid (NAA), and indole-3-butyric acid (IBA) (0, 10, 30, or 50 mg · L^−1^ of each exogenous hormone) immersion for 5, 10, 15, or 20 min was conducted. Callus initiation rate and rooting promotion rate were mainly affected by treatment time, root length increase by 6-BA concentration, and bud germination rate and plantlet formation rate by NAA concentration. The expected optimal combination for callus initiation rate was 50 mg · L^−1^ 6-BA + 0 mg · L^−1^ NAA + 30 mg · L^−1^ IBA + 10 min; for rooting promotion rate, it was 0–10 mg · L^−1^ 6-BA + 10 mg · L^−1^ NAA + 30 mg · L^−1^ IBA + 20 min; for bud germination rate, it was 50 mg · L^−1^ 6-BA + 0–10 mg · L^−1^ NAA + 0–30 mg · L^−1^ IBA + 20 min; and for seedling formation rate for No. 4, it was 10 mg · L^−1^ 6-BA + 10 mg · L^−1^ NAA + 0 mg · L^−1^ IBA + 20 min. Light microscopy image analysis revealed that a cluster of primordial cells was produced 40 days after cutting, and mastoid cells developed into peninsula cells in calli that were cultured for 50 days.

## Introduction

Chinese fir (*Cunninghamia lanceolata* [Lamb.] Hook) is one of the most economically important indigenous coniferous species in south China. Because of its fast growth, excellent timber quality, and pest and disease resistance, it has been widely planted since the 1980s, in tropical and subtropical areas. According to the State Forestry Administration of the People’s Republic of China^1^, Chinese fir plantations cover 1.10 × 10^7^ hm^−2^, and account for 21.35% of the total plantation area in China. Because of the increasing demand for timber products, the planting areas of Chinese fir plantations have expanded rapidly, from small hills to high mountains and from fertile sites to infertile areas^2^. Hence, the demand for the excellent cultivars has been increasing. Traditionally, plantation cultivars primarily come from seedlings from superior Chinese fir families. The major shortcoming of seed propagation is genetic recombination in the next generation, which results in offspring that have difficulty retaining the superior characteristics of the parent plants. Furthermore, seedlings from nursery stocks are insufficient to meet the huge production requirements of Chinese fir plantations^3, 4^. Asexual propagation technology is a prospective method for producing numerous cloned plantlets, which compared to seedlings, are more efficient, reliable, stable, and reproducible, and may ease the shortage of orchard seedlings. However, some problems with this technique still exist, such as low rooting rate and low callus initiation rate^5–7^. Therefore, in order to increase the number of high-yield Chinese fir plantations, cutting propagation, which has been widely used in other species, should be taken into consideration^8–10^.

Tissue culture is the science of growing plant tissues or organs that are isolated from the mother plant on artificial media. It can help meet the demand for more efficient horticultural and agriculture production^5, 6, 11^. Previous studies have been conducted on the establishment of an efficient tissue culture system for rapidly propagating Chinese fir; these have focused on callus induction and axillary bud and/or shoot differentiation and regeneration^12–14^. Wang *et al*.^12^ studied the germination of axillary buds and the proliferation of multiple shoots under different types and concentrations of plant growth regulators and reported that the best medium for the first generation was Murashige and Skoog medium (MS) + 1.0 mg · L^−1^ 6-Benzylaminopurine (6-BA) + 0.5 mg · L^−1^ 1-naphthaleneacetic acid (NAA); for proliferation, it was MS + 0.8 mg · L^−1^ 6-BA + 0.3 mg · L^−1^ NAA. Pang *et al*.^13^ investigated the effects of culture and the indole-3-butyric acid (IBA)/NAA combination ratio on root induction in tissue-cultured seedlings, and selected the optimal combination. However, most of these tissue-cultured materials were explanted from stem segments, and the propagation requirements of branch cuttings from superior mother clones are difficult to meet. Furthermore, tissue culture is expensive, requires the management of professional workers, and induction culture related to calli is difficult; indeed, they have been subcultured for no more than two or three generations^7, 15^.

Cutting propagation is commonly used in the commercial production of ornamental foliage crops, medical plants, and wood trees^16, 17^. Previous studies have focused on the effects of exogenous auxins on adventitious root formation and bud germination. It has been reported that the root development and bud germination of cutting segments depend on the species and cultivar, segment position, age of parent plants, and other factors^18, 19^; common root-promoting chemicals contain 6-BA, IBA, or NAA^20, 21^. Since the early 1990s, it has been reported that plantations that use stem cuttings taken from superior Chinese fir clones exhibit faster growth and better wood quality than those that use seedlings^22, 23^. Plantlets obtained from stem cuttings are characterized by rapid initial growth, and may increase the production of small- to medium-diameter Chinese fir plantations in a short-term rotation^24–26^. Cuttings are mainly taken from stems and branches, and the survival rate of cuttings taken from the base shoot is the highest, followed by cuttings from stems. Cuttings from old branches have the lowest survival rate^27^. Wu *et al*.^28^ reported that the rooting and germinating ability of lignified or semi-lignified stem cuttings sustainedly increased in sandy soil nursery bed. Zou *et al*.^29^ found that IBA and IAA of 50 μg · g^−1^ had significant promoting effects on the rooting rate of stem cuttings. However, in general, the quantity and quality of stem cuttings generated from superior mother clones is insufficient to meet the requirements for Chinese fir afforestation. Stock plants derived from leaf cuttings can produce more new cuttings than those derived from tissue culture and stem cutting. Leaf cuttings are simple and inexpensive, and are commonly used in the commercial production of vegetable and ornamental foliage crops^21, 30, 31^. For Chinese fir species, few previous studies have reported that plantlets were generated from leaf segment cuttings, and most have focused on the effects of cutting season, soil type, and hormone treatment on the morphological features of leaf cuttings of Chinese fir. The internal mechanism of bud germination and rooting growth generated from leaf cuttings require further study^32, 33^. Therefore, the establishment of a highly efficient propagation system for leaf cuttings of Chinese fir is urgently required.

In view of the shortage of Chinese fir nursery seedlings, leaf cuttings from three superior Chinese fir clones (No. 4, No. 6, and No. 7) were used in this study. We hypothesized that callus initiation rate, axillary bud germination, root growth, and plantlet survival rate would be affected by hormone concentration and treatment time. We also hypothesized that the responses of these variables would vary among asexual clones. Using an orthogonal L_16_(4)^4^ design (Table 1), the objectives of this study were (1) to investigate the effects of auxin treatment on root formation, axillary bud germination, and plantlet survival rate; and (2) to establish the optimum hormone combination for leaf cuttings taken from Chinese fir. This study provides a leaf-cutting method in order to promote its practical application in the nursery industry, and has important theoretical and practical significance in easing the shortage of Chinese fir nursery stock.

**Table 1:** L_16_(4)^4^ orthogonal design used in the study.

Test no.	(A) 6-BA (mg · L^−1^)		(B) NAA (mg · L^−1^)		(C) IBA (mg · L^−1^)		(D) Time (min)	
1	A_1_	0	B_1_	0	C_1_	0	D_1_	5
2	A_1_	0	B_2_	10	C_2_	10	D_2_	10
3	A_1_	0	B_3_	30	C_3_	30	D_3_	15
4	A_1_	0	B_4_	50	C_4_	50	D_4_	20
5	A_2_	10	B_1_	0	C_2_	10	D_3_	15
6	A_2_	10	B_2_	10	C_1_	0	D_4_	20
7	A_2_	10	B_3_	30	C_4_	50	D_1_	0
8	A_2_	10	B_4_	50	C_3_	30	D_2_	10
9	A_3_	30	B_1_	0	C_3_	30	D_4_	20
10	A_3_	30	B_2_	10	C_4_	50	D_3_	15
11	A_3_	30	B_3_	30	C_1_	0	D_2_	10
12	A_3_	30	B_4_	50	C_2_	10	D_1_	0
13	A_4_	50	B_1_	0	C_4_	50	D_2_	10
14	A_4_	50	B_2_	10	C_3_	30	D_1_	0
15	A_4_	50	B_3_	30	C_2_	10	D_4_	20
16	A_4_	50	B_4_	50	C_1_	0	D_3_	15

## Results

### Optimization of callus initiation rate

**Figure 1:**
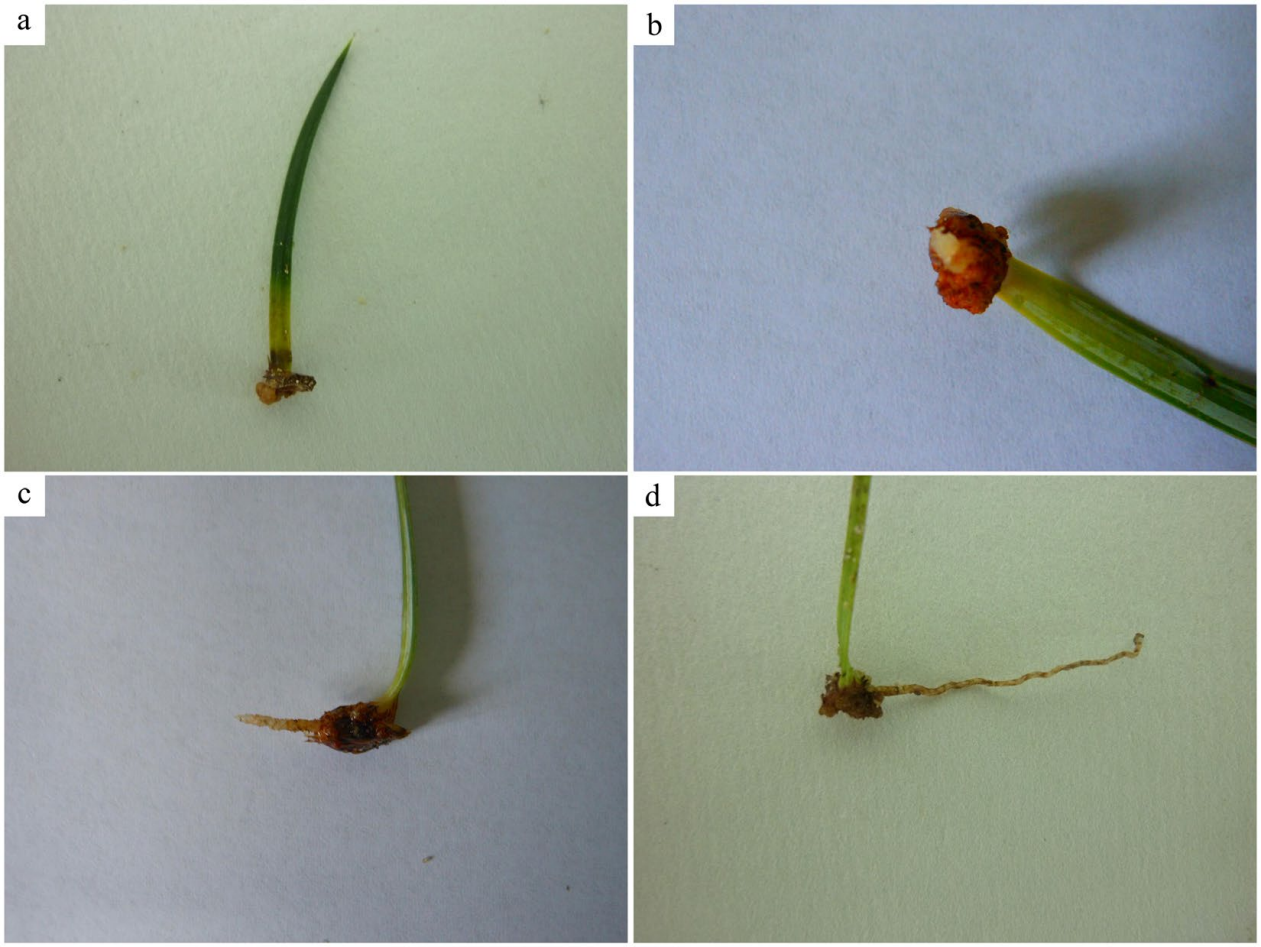
Callus and root initiation in a leaf cutting from Chinese fir. (**a**) Callus initiation 15 days after cutting; (**b**) adventitious root appears 50 days after cutting; (**c**) root growth at 60 days after cutting; (**d**) root growth at 90 days after cutting.

**Figure 2:**
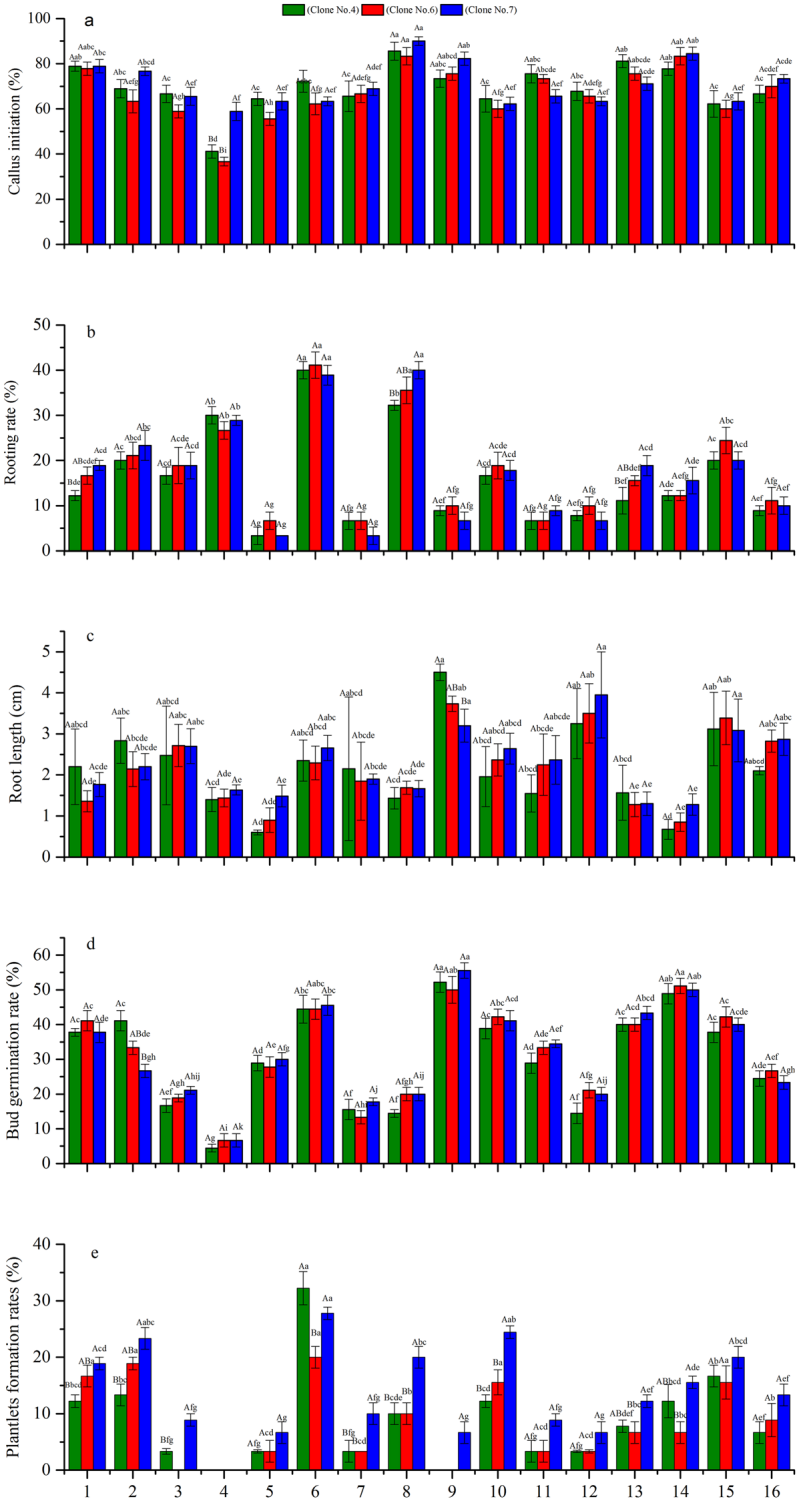
Effects of different hormone combinations of leaf cuttings taken from Chinese fir clones. Treatment numbers 1–16 are shown on the x-axis, and values for the three different clones are represented by the three different colored columns. (**a**) Callus initiation rates; (**b**) rooting rates; (**c**) root length; (**d**) bud germination rates; (**e**) plantlet formation rates. *Different lowercase letters* above the columns indicate significant difference between treatments for the same clone (*p* < 0.05). *Different capital letters* indicate significant difference between clones under the same treatment (*p* < 0.05). Values are means and the error bars represent standard error.

A milky white protuberance was observed at the leaf base 8 days after cutting, and callus initiation occurred 15 days after cutting (Fig. 1a). Needles without callus formation started to die. The callus initiation rate did not vary significantly among treatments and clones (*p* = 0.508 and 0.525, respectively) (Fig. 2a). The maximum callus initiation rates in clones No. 4, No. 6, and No. 7 were obtained under treatment 8 (A_2_B_4_C_3_D_2_) (85.5%, 83.3%, and 90.0%, respectively), and were significantly higher than the minimum callus initiation rate under treatment (41.1%, 36.7%, and 58.9%, respectively). According to the *R* values, the effects of hormone concentration and treatment time decreased in the following order: D > C > B > A, C > D > A > B, and C > D > B > A in No. 4, No. 6, and No. 7, respectively (Table 2). Treatment time and IBA were the most important determinants of callus initiation rate. Specifically, the optimal combination for callus initiation rate in the three clones was A_4_B_1_C_3_D_2_ (50 mg · L^−1^ 6-BA + 0 mg · L^−1^ NAA + 30 mg · L^−1^ IBA + 10 min).

**Table 2:** Range analysis of L_16_(4)^4^ test results for callus initiation rate.

Variables	Clone no. 4				Clone no. 6				Clone no. 7			
	A	B	C	D	A	B	C	D	A	B	C	D
*K*_1_	63.90	**74**.**45**	73.33	72.50	59.17	**71**.**11**	70.83	73.33	70.00	**73**.**89**	70.28	73.89
*K*_2_	71.93	70.83	65.83	**77**.**78**	66.94	67.22	61.11	**73**.**89**	71.39	71.67	66.67	**75**.**83**
*K*_3_	70.27	67.49	**75**.**83**	65.55	68.61	64.72	**75**.**28**	61.11	68.33	65.83	**80**.**56**	66.11
*K*_4_	**71**.**94**	65.28	63.07	62.22	**72**.**22**	63.89	59.72	58.61	**73**.**06**	71.39	65.28	66.95
*R*	8.04	9.17	12.76	15.56	13.06	7.22	15.56	15.28	4.72	8.06	15.28	9.72
Optimal level	A_4_	B_1_	C_3_	D_2_	A_4_	B_1_	C_3_	D_2_	A_4_	B_1_	C_3_	D_2_

The optimal level is in bold.

### Optimization of rooting rate

At 50 days after cutting, adventitious roots started appearing from the callus induction parts (Fig. 1b). Most needles could only form one root, but several needles formed more than one root (Fig. 1c). Rooting rate did not vary significantly among treatments and clones (*p* = 0.755 and 0.821, respectively). The maximum callus initiation rate in clones No. 4 and No. 6 was obtained under treatment 6 (A_2_B_2_C_1_D_4_), and in No. 7 under treatment 8 (A_2_B_2_C_1_D_4_) (Fig. 2b). According to the *R* values, the effects of hormone concentration and treatment time decreased in the following order: D > B > A > C, D > A > B > C, and D > A > B > C for No. 4, No. 6, and No. 7, respectively (Table 3). Treatment time was the most important determinant of rooting promotion rate. Specifically, the expected optimal combination for rooting promotion rate in No. 4 and No. 6 was A_2_B_2_C_3_D_4_ (10 mg · L^−1^ 6-BA + 10 mg · L^−1^ NAA + 30 mg · L^−1^ IBA + 20 min), and in No. 7 it was A_1_B_2_C_3_D_4_ (0 mg · L^−1^ 6-BA + 10 mg · L^−1^ NAA + 30 mg · L^−1^ IBA + 20 min).

**Table 3:** Range analysis of L_16_(4)^4^ test results for rooting rate.

Variables	Clone no. 4				Clone no. 6				Clone no. 7			
	A	B	C	D	A	B	C	D	A	B	C	D
*K*_1_	19.72	8.89	16.94	9.72	20.83	12.22	18.89	11.39	**22**.**50**	11.94	19.17	11.11
*K*_2_	**20**.**56**	**22**.**22**	12.78	17.50	**22**.**50**	**23**.**33**	15.56	19.72	21.39	**23**.**89**	13.33	22.78
*K*_3_	10.00	12.50	**17**.**50**	11.39	11.39	14.17	**19**.**17**	13.89	10.00	12.78	**20**.**28**	12.50
*K*_4_	13.06	19.72	16.11	**24**.**72**	15.83	20.83	16.94	**25**.**56**	16.11	21.39	17.22	**23**.**61**
*R*	10.55	13.33	4.72	15.00	11.11	9.17	3.61	14.17	12.50	11.95	6.95	11.67
Optimal level	A_2_	B_2_	C_3_	D_4_	A_2_	B_2_	C_3_	D_4_	A_1_	B_2_	C_3_	D_4_

The optimal level is in bold.

### Optimization of root length growth

One-way ANOVA indicated that root length growth varied significantly among the treatments and clones (*p* = 0.000 and 0.001, respectively). The maximum root length in clone No. 4 was obtained under treatment 9 (A_3_B_1_C_3_D_4_), in No. 6 under treatment 12 (A_3_B_4_C_2_D_1_), and in No. 7 under treatment 15 (A_4_B_3_C_2_D_4_) (Fig. 2c). According to the *R* values, the effects of hormone concentration and treatment time decreased in the following order: A > D > B > C, A > C > B > D, and A > C > D > B for No. 4, No. 6, and No. 7, respectively (Table 4). Treatment with 6-BA was the most important determinant of root growth. Specifically, the optimal combination for root growth in clone No. 4 and No. 6 was A_3_B_3_C_2_D_4_ (30 mg · L^−1^ 6-BA + 30 mg · L^−1^ NAA + 10 mg · L^−1^ IBA + 20 min), and in No. 7 it was A_3_B_4_C_2_D_4_ (30 mg · L^−1^ 6-BA + 50 mg · L^−1^ NAA + 10 mg · L^−1^ IBA + 20 min).

**Table 4:** Range analysis of L_16_(4)^4^ test results for root length.

Variables	Clone no. 4				Clone no. 6				Clone no. 7			
	A	B	C	D	A	B	C	D	A	B	C	D
*K*_1_	2.23	2.22	2.05	2.07	1.91	1.82	2.18	1.89	2.08	1.94	2.41	2.22
*K*_2_	1.63	1.95	**2**.**45**	1.85	1.68	1.91	**2**.**48**	1.84	1.93	2.19	**2**.**68**	1.88
*K*_3_	**2**.**82**	**2**.**32**	2.27	1.78	**2**.**96**	**2**.**55**	2.25	2.20	**3**.**04**	2.51	2.21	2.42
*K*_4_	1.87	2.05	1.77	**2**.**84**	2.09	2.36	1.73	**2**.**71**	2.13	**2**.**53**	1.87	**2**.**64**
*R*	1.18	0.37	0.50	1.06	1.28	0.73	0.51	0.87	1.11	0.59	0.81	0.76
Optimal level	A_3_	B_3_	C_2_	D_4_	A_3_	B_3_	C_2_	D_4_	A_3_	B_4_	C_2_	D_4_

The optimal level is in bold.

### Optimization of bud germination rate

**Figure 3:**
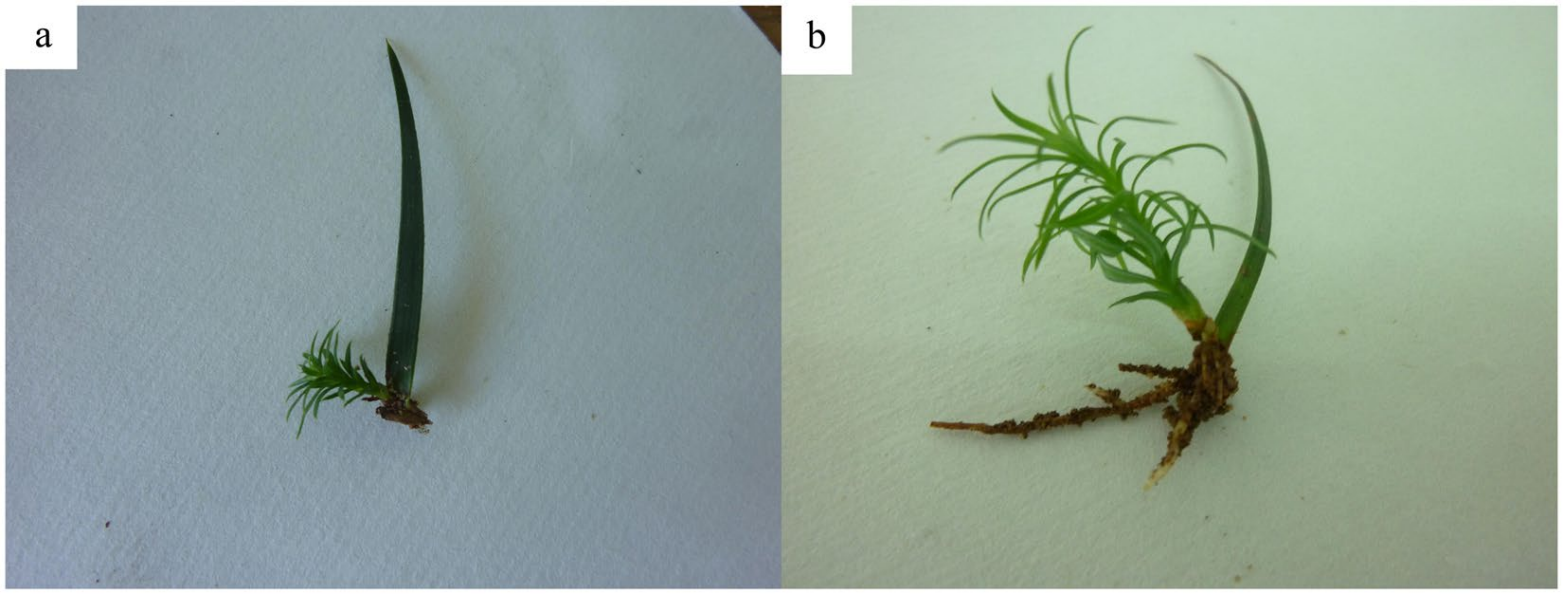
Axillarybudand seedling growth in a leaf cutting from Chinese fir. (**a**) Axillary bud appears 60 days after cutting; (**b**) plantlet development 90 days after cutting.

Most leaf cuttings showed axillary buds at 50 days after cutting (Fig. 3a). Bud germination rate did not vary significantly among the treatments and clones (*p* = 0.284 and 0.782, respectively). The maximum bud germination rate in clones No. 4 and No. 7 was obtained under treatment 9 (A_3_B_1_C_3_D_4_), and in No. 6 it was obtained under treatment 14 (A_4_B_2_C_3_D_1_) (Fig. 2d). According to the *R* values, the effects of hormone concentration and treatment time decreased in the following order: B > A > C > D in all three clones (Table 5). NAA was the most important determinant of bud germination rate. Specifically, the optimal combination for bud germination rate in No. 4 and No. 6 was A_4_B_2_C_1_D_4_ (50 mg · L^−1^ 6-BA + 10 mg · L^−1^ NAA + 0 mg · L^−1^ IBA + 20 min), and in No. 7 it was A_4_B_1_C_3_D_4_ (50 mg · L^−1^ 6-BA + 0 mg · L^−1^ NAA + 30 mg · L^−1^ IBA + 20 min).

**Table 5:** Range analysis of L_16_(4)^4^ test results for bud germination rate.

Variables	Clone no. 4				Clone no. 6				Clone no. 7			
	A	B	C	D	A	B	C	D	A	B	C	D
*K*_1_	25.00	39.72	**33**.**89**	29.17	25.00	39.72	**36**.**39**	31.67	23.06	**41**.**67**	35.28	31.39
*K*_2_	25.83	**43**.**33**	31.11	31.11	26.39	**42**.**78**	31.11	31.67	28.33	40.83	29.17	31.11
*K*_3_	33.61	24.72	33.06	27.22	36.67	26.95	35.00	28.89	37.78	28.33	**36**.**67**	28.89
*K*_4_	**37**.**78**	14.44	24.72	**34**.**72**	**40**.**00**	18.61	25.56	**35**.**83**	**39**.**17**	17.50	27.22	**36**.**94**
*R*	12.78	28.89	9.17	7.50	15.00	24.17	10.83	6.94	16.11	24.17	9.44	8.06
Optimal level	A_4_	B_2_	C_1_	D_4_	A_4_	B_2_	C_1_	D_4_	A_4_	B_1_	C_3_	D_4_

The optimal level is in bold.

### Optimization of plantlet formation rate

Plantlet growth started 70 days after cutting (Fig. 3b). Plantlet formation rate varied significantly among the treatments (*p* = 0.002), but not among the clones (*p* = 0.881). The maximum plantlet formation rate in the three clones was obtained under treatment 6 (A_2_B_2_C_1_D_4_) (Fig. 2e). According to the *R* values, the effects of hormone concentration and treatment time decreased in the following order: B > C > A > D, B > C > A > D, and B > A > C > D (Table 6). NAA was the most important determinant of bud germination rate. Specifically, the optimal combination for plantlet formation rate in No. 4 was A_2_B_2_C_1_D_4_ (10 mg · L^−1^ 6-BA + 10 mg · L^−1^ NAA + 0 mg · L^−1^ IBA + 20 min), in No. 6 it was A_4_B_2_C_1_D_2_ (50 mg · L^−1^ 6-BA + 10 mg · L^−1^ NAA + 0 mg · L^−1^ IBA + 10 min) and No. 7 it was A_2_B_2_C_1_D_2_ (10 mg · L^−1^ 6-BA + 10 mg · L^−1^ NAA + 0 mg · L^−1^ IBA + 10 min).

**Table 6:** Range analysis of L_16_(4)^4^ test results for plantlet formation rate.

Variables	Clone no. 4				Clone no. 6				Clone no. 7			
	A	B	C	D	A	B	C	D	A	B	C	D
*K*_1_	7.22	5.83	**13**.**61**	7.78	7.22	6.67	**12**.**22**	7.50	12.78	11.11	**17**.**22**	12.78
*K*_2_	**12**.**22**	**17**.**50**	9.17	8.61	9.17	**15**.**28**	10.28	**9**.**72**	**16**.**11**	**22**.**78**	14.17	**16**.**11**
*K*_3_	4.72	6.67	6.39	6.39	5.55	5.56	4.17	6.94	11.67	11.95	12.78	13.33
*K*_4_	10.83	5.00	5.83	**12**.**22**	**9**.**44**	5.55	6.39	8.89	15.28	10.00	11.67	13.61
*R*	7.50	12.50	7.78	5.84	3.89	9.72	8.06	2.78	4.45	12.78	5.56	3.33
Optimal level	A_2_	B_2_	C_1_	D_4_	A_4_	B_2_	C_1_	D_2_	A_2_	B_2_	C_1_	D_2_

The optimal level is in bold.

### Light microscopy imaging

**Figure 4:**
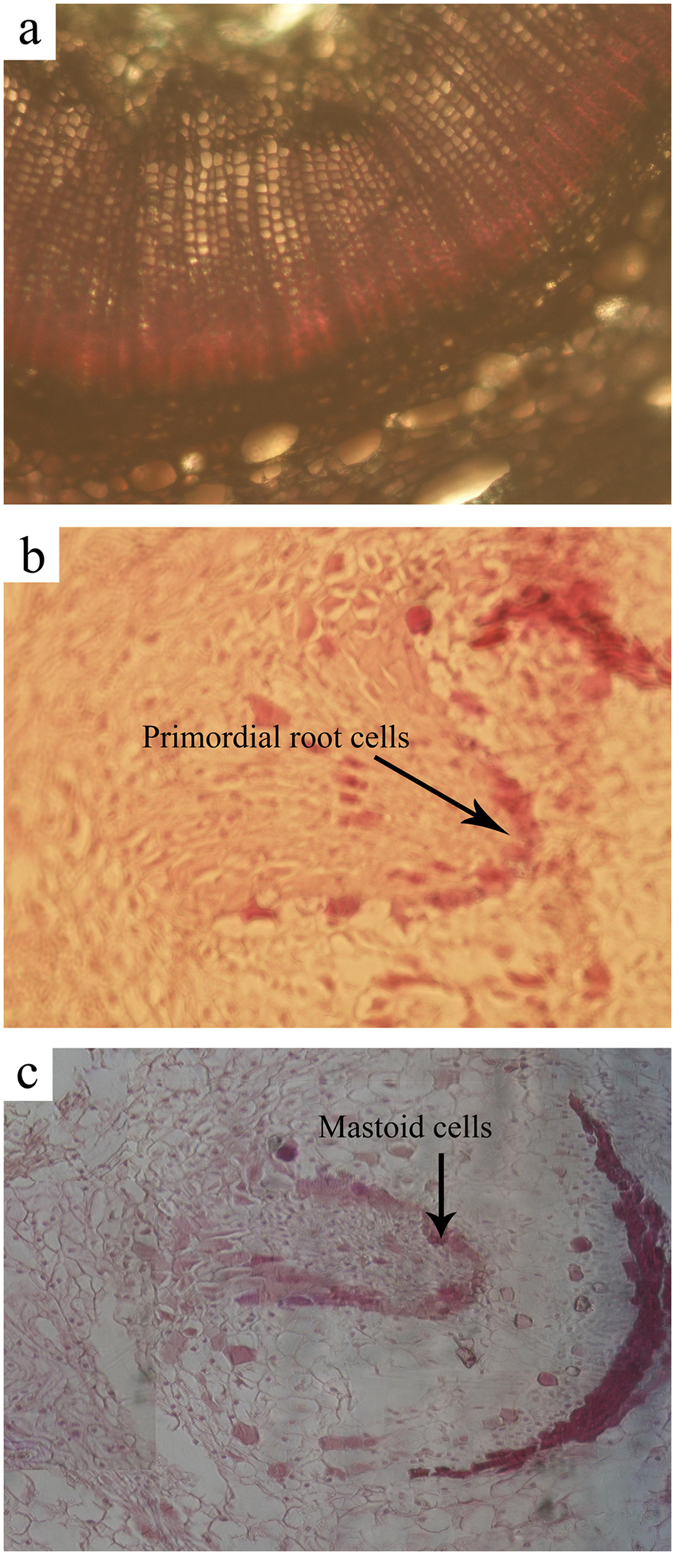
Light microscopy images of rooting from a leaf cutting taken from Chinese fir at 400× magnification. (**a**) Leaf segment before cutting from the mother plant; (**b**) primordial root originating from calli 40 days after cutting; (**c**) expansion and extension of primordial cells and peninsula tissues in callus initiation 50 days after cutting.

An anatomical observation of the transverse section of the leaf segment before cutting indicated that there was no potential pre-primordial root development in the cortex, phloem, xylem, cambium, or pith (Fig. 4a), suggesting that adventitious root formation is induced from primordial root cells. A cluster of primordial cells was produced 40 days after cutting in the calli, which became dark with dense cytoplasm (Fig. 4b). With the expansion and extension of the primordial cells, a cluster of mastoid cells developed into peninsula tissue in the calli 50 days after cutting (Fig. 4c).

## Discussion

Our results demonstrate that auxin and treatment time had large effects on root formation, axillary bud germination, and plantlet survival rate, which is consistent with our hypotheses and previous results^34^. Previous studies have demonstrated that exogenous application of auxins has positive effects on rooting percentage and numbers of roots^10, 35–37^. Application of 6-BA, IBA, and NAA has often been recommended for promoting rooting in cutting propagation^10, 38^. For example, Ludwig-Müller^10^ reported IAA and IBA had positive promotion effects on primary root growth and lateral root elongation in *Pueraria lobata* at 0.1 μmol · L^−1^; however, they had negative inhibition effects at 2.5 μmol · L^−1^. Yang *et al*.^39^ reported that exogenous IAA, IBA, or NAA auxins stimulate hairy roots growth in *Nepeta cataria*, and IBA at 0.5 mg · L^−1^ produced the highest levels of growth. Similarly, exogenous IAA or NAA increases biomass and lobeline production in hairy roots of *Lobelia inflata* ^40^. Before our formal experiment was conducted, according to results from Guizhou Agriculture College and Jiangxi Forestry Institute Leaf Cutting Study Groups of Chinese fir^41, 42^, an L_16_(4)^4^ orthogonal test design was carried out with auxin concentrations of 6-BA, NAA, and IBA at 0 mg · L^−1^, 50 mg · L^−1^, 100 mg · L^−1^ and 150 mg · L^−1^. The immersion times were 10 min, 30 min, 60 min, and 120 min. The total number of replicates was 480 (16 treatments × 30 leaf cuttings per replicate). In this preliminary experiment, we found that 6-BA was most important for stimulating callus initiation rate, and that callus initiation rate reached a maximum value at 50 mg · L^−1^ of 6-BA and NAA, respectively. Callus initiation rate greatly decreased with auxin concentration and increasing time (data not shown). Based on this preliminary study the maximum concentration of hormone was set at 50 mg · L^−1^. In this study, we found that root length and plantlet formation rate were significantly affected by the orthogonal treatments (*p* = 0.000 and 0.002, respectively); however, callus initiation rate, rooting rate, bud germination rate, and plantlet formation rate did not significantly differ among Chinese fir clones (Fig. 3). The range analysis indicated that IBA and 6-BA were the determinants of callus initiation rate and root growth (rooting rate and average root length), respectively. NAA was the determinant of bud germination rate and plantlet formation rate (Tables 2, 3, 4, 5 and 6). For superior Chinese fir leaf cuttings, the expected optimal combination for rooting percentage was 0–10 mg · L^−1^ 6-BA, 10 mg · L^−1^ NAA, and 30 mg · L^−1^ IBA, with an immersion time of 20 min. The optimal combination for root length growth was 30 mg · L^−1^ 6-BA, 30–50 mg · L^−1^ NAA, and 10 mg · L^−1^ IBA, with an immersion time of 20 min. However, plant genotype, plant age, auxin type and concentration, and cutting date may influence root initiation^16, 43^. Su *et al*.^44^ found that rooting rate reached a maximum (74.15%) at a combination of 300 mg · L^−1^ NAA and 40 mg · L^−1^ IBA. Singh *et al*.^34^ reported that NAA (2,000 mg · L^−1^) was more effective at inducing rooting than IBA in *Dendrocalamus asper* stem cuttings. Zeng *et al*.^45^ reported that when softwood cuttings of tree peony were immersed in 150 mg · L^−1^ IBA for 16 h, the rooting percentage was as high as 93%.

Treatment method is another important cause of variation in rooting success. Many people use the basal quick-dip and immersion methods, whereas others use the leaf-spraying application method. The auxin concentration is higher with the quick-dip method (1,000–2,000 mg · L^−1^ IBA) than with the immersion method (150 mg · L^−1^ IBA)^16, 45^. Blythe *et al*.^17^ compared the rooting and initial shoot growth of stem cuttings of four tropical ornamentals using different auxin treatments. For *Gardenia augusta* ‘Radicans’, the best rooting was obtained using a spray containing 123.0 mg · L^−1^ IBA + 67.1 mg · L^−1^ NAA after insertion into the rooting substrate; but for *Ficus benjamina*, a basal quick-dip in auxin or spay applications of auxin did not provide any improvement in rooting^17^. In our study, the basal leaves were immersed in the Plant Growth Regulators at different auxin combinations for 20 min prior to leaf cutting, which greatly improved the rooting and root growth of Chinese fir leaf cuttings.

We found that adventitious roots were produced from primordial cells in the calli of the leaf cuttings. However, some researchers have found that primordial cell formation in Chinese fir stem cuttings mainly originates in the cortex, and rarely in the calli^46, 47^. That is why the rooting rate was higher (most ca. 90%) for cutting cultivars generated from stems than those generated from leaves (only ca.16%) in our study. The anatomic process of shoot rooting in our study showed there were no latent root primordia in the shoot at the earlier stage, and the induced root primordia originated and differentiated from the calli of the basal leaves 40 days after cutting (Fig. 4a–c). For poplar, willow, and mume species, root initiation from stem cuttings differentiated from cortical parenchymal cells, and there was root primordium in the shoot, which resulted in a high rooting rate after cutting^48–52^. We found that primordial cell differentiation in the calli started 40 days after cutting (Figs 1c and 4b), while primordial cells grew into mastoid cells after 7 to 10 days of culture. The initiation and differentiation of primordium cells is affected by endogenous IBA^46, 49^. Zeng *et al*.^49^ reported that root initiation is promoted by exogenous IBA application (100 mg · L^−1^); exogenous IBA application in tree peony shoots results in an increase in endogenous IAA, which triggers cambium cell differentiation due to auxin sensitivity. IAA has been suggested as a prime trigger for root initiation^53^, but Guo *et al*.^16^ found that endogenous IAA content decreased with time and had a negative effect on rooting potential. They reported that zeatin riboside (ZR) content was positively related to rooting response in *Paeonia*. For Chinese fir species, the main problem with the propagation of leaf cuttings is the low rooting rate; therefore, in order to successfully propagate superior Chinese fir clone leaf cuttings, a variety of factors should be taken into account, including auxin type, concentration, combination ratio, plant genotype, and leaf age. In general, our results provide a basis for the leaf cutting propagation of Chinese fir. Future studies should investigate the relationship between endogenous hormones and exogenously applied auxins and leaf cutting time in order to improve the leaf propagation of Chinese fir clones.

## Conclusion

The results of our study may ease the shortage of Chinese fir orchard seedlings. We found that immersing leaves in exogenous auxins before cutting had positive effects on bud germination and adventitious root initiation, but the effects varied greatly among different clones, supporting our hypotheses. Based on orthogonal L_16_(4)^4^ design, the range analysis results showed that the expected optimal combination for callus initiation was 50 mg · L^−1^ 6-BA + 0 mg · L^−1^ NAA + 30 mg · L^−1^ IBA + 10 min, and for root formation, it was 0–10 mg · L^−1^ 6-BA + 10 mg · L^−1^ NAA + 30 mg · L^−1^ IBA + 20 min. Roots were produced from a cluster of primordial cells in callus tissue instead of in the cortex, and root initiation from leaf cuttings was later than that from stem cuttings, which increases the difficulty of Chinese fir leaf cutting. The main issue is whether species can readily produce adventitious roots. Therefore, to improve rooting and root growth from leaf cuttings, the way in which exogenous auxins (e.g., NAA and 6-BA) influence endogenous hormones (e.g., IBA, IAA, and ZR) should be investigated in future studies.

## Materials and Methods

### Experimental materials

Cutting propagation material was taken from superior Chinese fir (*Cunninghamia lanceolata*) clones No. 4, No. 6, and No. 7, which were provided by the Chinese Fir Engineering Technology Research Center, Fuzhou, China. The clones were two years old, and the leaves were one year old and taken from the first lateral branch. Leaf cuttings were taken with a relatively short piece (2–3 mm) of shoot tissue below an axillary bud using a cutting blade on a sunny morning. Thus, the leaf cuttings had a concave profile with axillary buds and xylem, in order to improve axillary shoot development. The bottom halves of the cuttings were immersed in different hormone combinations for different periods (Table 1). The leaves were trimmed by about two-thirds in clean and moist beds. The spacing within and between rows was 1 × 10 cm. The cutting date was August 21, 2011.

### Hormone combination design

An orthogonal L_16_(4)^4^ design was used. Thus, 16 combinations of different hormones (6-Benzylaminopurine [6-BA], 1-naphthaleneacetic acid [NAA], and indole-3-butyric acid [IBA]) concentrations (0, 10, 30, or 50 mg · L^−1^) and immersion periods (5, 10, 15, or 20 min) were tested in a single-factor test (Table 1). The total leaf number for each treatment was 90 (3 replicates × 30 leaves per replicate).

### Greenhouse management

The leaf cuttings were planted in a greenhouse on the campus of Fujian Agriculture and Forestry University, Fuzhou, China. The seedbed medium was homogeneous river sand, which was disinfected with 0.3% (w/v) KMnO_4_ before leaf cutting. A shade net was placed over the greenhouse to prevent excessive heat, and to regulate light conditions to a 30–50% light transmittance rate. Air temperature in the greenhouse was maintained at approximately 30 °C, and the relative humidity was about 85%. During the study period, cuttings were maintained under an intermittent mist using an overhead boom automatic irrigation system. Misting was conducted for 15 s every 30 min during the day, in order to keep the foliage fresh. For controlling fungal disease, 50% carbendazol (diluted 800×) was sprayed weekly during the study period.

### Light microscopy imaging

The root structure of the leaf cuttings was observed using the modified paraffin section method^35^. First, excised segments of stem and leaf cuttings were immediately placed in an FAA fixing solution (90 mL of 70% ethanol + 5 mL of acetic acid + 5 mL of formalin) for 24 h at room temperature. Subsequently, the tissues were rinsed three times (70% ethanol, 1 h), dehydrated (85% ethanol, 1 h; 95% ethanol, 1 h; 100% ethanol, 30 min; 100% ethanol, 30 min), made transparent (1/2 ethanol:1/2 xylene, 1 h; xylene, 30 min; xylene, 30 min), waxed (1/2 xylene:1/2 paraffin, 1 h at 37 °C; paraffin, 30 min and paraffin, 30 min at 62 °C), and embedded in paraffin. Finally, the paraffin blocks were cut to 10 µm using a microtome and deparaffinized (xylene, 5 min; xylene, 5 min;), rehydrated (50% ethanol + 50% xylene; 100% ethanol; 100% ethanol; 85% ethanol; 70% ethanol; 50% ethanol; 30% ethanol; distilled water, 5 min per step), dyed (stained for 1 h with 1% safranin; 35% ethanol, 5 min; 50% ethanol, 5 min; 70% ethanol, 3 min; 80% ethanol, 3 min; 100% ethanol, 1 min; 100% ethanol, 3 min; 1/2 100% ethanol + 1/2 xylene, 5 min; xylene, 5 min), and sealed using Permount Mounting Medium prepared with 3:1 rhamsan gum: xylene). Images were taken using a Ti-S200 inverted fluorescence microscope (Nikon, Japan). Before cutting, we recorded a transverse-section leaf light microscopy image, and a leaf light microscopy image was recorded every two weeks after cutting.

### Measurements and statistical analysis

After 12 weeks, the cuttings were evaluated for callus initiation rate, rooting formation rate, root growth, bud germination rate, and plantlet formation rate. The callus initiation rate was calculated as the number of callus initiations divided by the total number of leaf cuttings; rooting formation rate as the number of leaf cuttings with root formation divided by the total number of leaf cuttings; average root length as the total length divided by the total number of leaf cuttings with root formation; bud germination rate as the number of bud germinations divided by the total number of leaf cuttings; and plantlet formation rate as the number of leaf cuttings with both bud germination and root formation divided by the total number of leaf cuttings. All data were analyzed using the SPSS statistical package (SPSS 17.0, SPSS Inc., IL, USA). We conducted one-way analysis of variance (ANOVA) with a least significant difference (LSD) multi-comparison test (*p* < 0.05) to determine whether the callus initiation rate, rooting formation rate, average root length, bud germination rate, and plantlet formation rate were significantly different among the orthogonal treatments. We also compared the values for different Chinese fir clones using one-way ANOVA (LSD test, *p* < 0.05). Furthermore, we conducted a range analysis to test the optimal level for Chinese fir leaf cuttings. The equation used was $R=\,{\rm{\max }}\,\{{K}_{i}^{X}\}-\,{\rm{\min }}\,\{{K}_{i}^{X}\}$, where ${K}_{i}^{X}$ is the average of the variables A, B, C and D at the 1, 2, 3, and 4 level, respectively; *X* is A, B, C, or D; and *i* is 1, 2, 3, or 4.
